# Metabolic syndrome and the plasma proteome: from association to causation

**DOI:** 10.1186/s12933-021-01299-2

**Published:** 2021-05-20

**Authors:** Mohamed A. Elhadad, Rory Wilson, Shaza B. Zaghlool, Cornelia Huth, Christian Gieger, Harald Grallert, Johannes Graumann, Wolfgang Rathmann, Wolfgang Koenig, Moritz F. Sinner, Kristian Hveem, Karsten Suhre, Barbara Thorand, Christian Jonasson, Melanie Waldenberger, Annette Peters

**Affiliations:** 1 Research Unit Molecular Epidemiology, Helmholtz Zentrum München, German Research Center for Environmental Health, Neuherberg, Germany; 2 Institute of Epidemiology, Helmholtz Zentrum München, German Research Center for Environmental Health, Neuherberg, Germany; 3 German Research Center for Cardiovascular Disease (DZHK), Partner site Munich Heart Alliance, Munich, Germany; 4 Weill Cornell Medicine-Qatar, Education City, PO Box 24144, Doha, Qatar; 5 German Center for Diabetes Research (DZD), München-Neuherberg, Ingolstädter Landstr. 1, 85764 Neuherberg, Germany; 6 Biomolecular Mass Spectrometry, Max Planck Institute for Heart and Lung Research, Ludwigstrasse 43, 61231 Bad Nauheim, Germany; 7 The German Centre for Cardiovascular Research (DZHK), Partner Site Rhine-Main, Max Planck Institute for Heart and Lung Research, Bad Nauheim, Germany; 8 Institute of Biometrics and Epidemiology, German Diabetes Center, Leibniz Center for Diabetes Research at Heinrich-Heine-University Düsseldorf, Düsseldorf, Germany; 9 Deutsches Herzzentrum München, Technische Universität München, Munich, Germany; 10 Institute of Epidemiology and Medical Biometry, University of Ulm, Ulm, Germany; 11 Department of Medicine I, University Hospital Munich, Ludwig-Maximilians-University, Munich, Germany; 12 K.G. Jebsen Center for Genetic Epidemiology, Department of Public Health, NTNU - Norwegian University of Science and Technology, Trondheim, Norway; 13 HUNT Research Center, Department of Public Health, NTNU - Norwegian University of Science and Technology, Levanger, Norway; 14 Chair of Epidemiology, Institute for Medical Information Processing, Biometry and Epidemiology, Medical Faculty, Ludwig-Maximilians-Universität München, Munich, Germany

**Keywords:** Metabolic syndrome, Proteomics, Blood proteins, Mendelian randomization analysis, Diabetes mellitus, type 2, Cardiovascular disease, Risk factors

## Abstract

**Background:**

The metabolic syndrome (MetS), defined by the simultaneous clustering of cardio-metabolic risk factors, is a significant worldwide public health burden with an estimated 25% prevalence worldwide. The pathogenesis of MetS is not entirely clear and the use of molecular level data could help uncover common pathogenic pathways behind the observed clustering.

**Methods:**

Using a highly multiplexed aptamer-based affinity proteomics platform, we examined associations between plasma proteins and prevalent and incident MetS in the KORA cohort (n = 998) and replicated our results for prevalent MetS in the HUNT3 study (n = 923). We applied logistic regression models adjusted for age, sex, smoking status, and physical activity.

We used the bootstrap ranking algorithm of least absolute shrinkage and selection operator (LASSO) to select a predictive model from the incident MetS associated proteins and used area under the curve (AUC) to assess its performance. Finally, we investigated the causal effect of the replicated proteins on MetS using two-sample Mendelian randomization.

**Results:**

Prevalent MetS was associated with 116 proteins, of which 53 replicated in HUNT. These included previously reported proteins like leptin, and new proteins like NTR domain-containing protein 2 and endoplasmic reticulum protein 29. Incident MetS was associated with 14 proteins in KORA, of which 13 overlap the prevalent MetS associated proteins with soluble advanced glycosylation end product-specific receptor (sRAGE) being unique to incident MetS. The LASSO selected an eight-protein predictive model with an (AUC = 0.75; 95% CI = 0.71–0.79) in KORA.

Mendelian randomization suggested causal effects of three proteins on MetS, namely apolipoprotein E2 (APOE2) (Wald-Ratio = − 0.12, Wald-p = 3.63e−13), apolipoprotein B (APOB) (Wald-Ratio = − 0.09, Wald-p = 2.54e−04) and proto-oncogene tyrosine-protein kinase receptor (RET) (Wald-Ratio = 0.10, Wald-p = 5.40e−04).

**Conclusions:**

Our findings offer new insights into the plasma proteome underlying MetS and identify new protein associations. We reveal possible casual effects of APOE2, APOB and RET on MetS. Our results highlight protein candidates that could potentially serve as targets for prevention and therapy.

**Supplementary Information:**

The online version contains supplementary material available at 10.1186/s12933-021-01299-2.

## Background

The metabolic syndrome (MetS) is a constellation of risk factors significantly increasing the risk of type 2 diabetes (T2D) and cardiovascular diseases (CVD) like coronary artery disease (CAD), stroke and heart failure [1, 2]. The respective risk factors are increased waist circumference, hypertriglyceridemia, reduced high-density lipoprotein, hyperglycemia and increased blood pressure. The prevalence of MetS has been steadily increasing in recent decades in conjunction with the obesity pandemic, driven by surplus eating and a sedentary lifestyle [3, 4]. It is estimated that 25% of adults worldwide have MetS, causing significant financial impact on healthcare systems [5].

Since its conception, the nature of MetS has been under debate [6–9]. However, most researchers agree that the clustering of the above mentioned risk factors is more frequent than could be attributed to chance alone [6–9]. In the center of the debate is MetS’ pathogenesis, which remains in the hypothesis stage. Suggested common driving pathogenic pathways include visceral adiposity and insulin resistance with subsequent dyslipidemia and subclinical inflammation [6]. While the suggested pathways help partly explain the clustering of risk factors and increased risk in some patients, they fail to explain the lack or incomplete clustering of those risk factors in others.

Recently, the introduction of omics data into CVD research has helped uncover molecular pathophysiological players, an example being the identification of PCSK9 as a drug target through genetic studies of CAD [10]. Omics studies with regard to cardio-metabolic risk factors have also been informative. Using the UK-Biobank data, a recent genetic study of MetS identified loci that are common to all MetS components as well as loci that are unique to the syndrome, i.e. not associated with the components themselves [11].

Proteomics, the study of proteins, can provide insight into the downstream players of genetics in the molecular pathogenic pathway of MetS and identify predictive biomarkers or targets for drug development. Enabled by advances in proteomics, studies with MetS have expanded from single protein to multi-protein investigations, the largest to date featuring 249 proteins [12]. Reported protein associations with MetS include adipokines like leptin and adiponectin (ADIPOQ), liver secreted proteins like sex hormone binding globulin (SHBG) and inflammatory markers like C-reactive protein, tumor necrosis factor alpha and complement system proteins [13, 14]. These proteins indicate functional links to MetS-defining features such as insulin resistance and visceral adiposity, and help explain the increased risk of complications, like CVD, in MetS patients.

In the present study, we use a highly multiplexed, aptamer-based, affinity proteomics platform (SOMAscan™) to assess the association between 1095 blood plasma proteins and prevalent and incident MetS in the KORA cohort, and replicate our results in the HUNT study. The proteins assessed by the SOMAscan platform have been selected to represent markers of a broad range of biological pathways and tissue specific processes. We investigate the use of these proteins as biomarkers and explore their potential causal effects using two-sample Mendelian randomization (MR) [15].

## Methods

### Study populations

#### KORA cohort

The KORA study (Cooperative health research in the Region of Augsburg) is a population-based cohort study from Augsburg, southern Germany. The study was approved by the ethics committee of the Bavarian Medical Association. Written informed consent was obtained from each participant. We used KORA-F4 (conducted 2006–2008) for cross-sectional analysis of prevalent MetS and its follow-up survey KORA-FF4 (conducted 2013–2014) for the prospective analysis of incident MetS (mean follow-up time = 6.5, SD = 0.5 years). For both surveys, detailed clinical and demographic information was collected, as was peripheral blood for later omics analyses. Details on the KORA cohort have been previously published [16]. A random subsample of 1000 individuals was selected from the already deeply phenotyped KORA-F4 study participants for proteomics measurement using the SOMAscan platform. One sample was excluded because it failed SOMALogic quality control and one participant was excluded due to the lack of sufficient information to define MetS leaving 998 participants for the final cross-sectional analysis. For the follow-up analysis, 371 participants with prevalent MetS and four participants lacking sufficient information to define incident MetS were excluded leaving 623 participants for analysis.

#### HUNT cohort

The Nord-Trøndelag Health Study (HUNT) is a population-based cohort from Nord-Trøndelag County in Norway. We used the HUNT3 survey (performed 2006–2008, N = 1017 with proteomics measurements) for the replication of the KORA study cross-sectional results. The HUNT study collected detailed socio-demographic and clinical information for all participants [17]. Ten samples failed SOMALogic quality control and were excluded from further analyses. Moreover, fourteen participants were excluded due to a lack of sufficient information to define MetS and an additional 70 participants were excluded due to missing information for the covariates smoking status and physical activity, leaving 923 participants for the final cross-sectional analysis.

### Proteomics measurement

The aptamer based SOMAscan platform was used to quantify proteins in both cohorts. Details on the platform [18] and its application to the KORA cohort have been described before [19]. In brief, each aptamer was selected to have high affinity toward a specific protein. Plasma was incubated with the aptamer mix and then exposed to multiple washing steps in the form of 2 bead-based immobilization steps to eliminate unbound or unspecifically bound aptamers and proteins. Finally, aptamers were eluted from the proteins and quantified as proxies to protein concentration by hybridization to custom arrays of aptamer-complementary oligonucleotides. The resulting raw intensities were processed with the help of standard samples included on each plate using a data analysis workflow consisting of hybridization normalization, median signal normalization and signal calibration to control for inter-plate differences [18]. The raw intensities are reported as relative florescence units.

Fasting plasma samples from the KORA study were sent to SomaLogic Inc. (Boulder Colorado, USA) for analysis [19]. Of the 1129 SOMAmer aptamers (SOMAscan assay V3.2) 29 failed SOMAscan quality control. We additionally removed five aptamers as recommended by the SOMAscan assay change log issued on December 22, 2016, leaving 1095 aptamers for analysis. For replication, we used only the HUNT aptamers that passed quality control [20].

### MetS definition in KORA

MetS was defined according to the harmonized definition by Alberti et al. [21] by the presence of three or more of the following criteria: (1) waist circumference ≥ 94 cm in men or ≥ 80 cm in women; (2) fasting serum triglycerides ≥ 150 mg/dl or drug treatment for elevated triglycerides (fibrates); (3) serum high density lipoprotein cholesterol (HDL) < 40 mg/dl in men or < 50 mg/dl in women or drug treatment for reduced HDL (fibrates); (4) systolic blood pressure ≥ 130 mmHg or diastolic blood pressure ≥ 85 mmHg or treatment with antihypertensive medication; (5) fasting serum glucose level ≥ 100 mg/dl or intake of antidiabetic medication.

### MetS definition in HUNT

The same definition was used for HUNT with some differences due to the unavailability of fasting measurements and information on drug treatment for elevated triglycerides or reduced HDL. For defining lipid components, we applied the cut-off levels suggested by Driver et al. for the diagnosis of metabolic syndrome using non-fasting lipid measurements [22]. For defining the low HDL component of MetS, we applied the same cut-off levels as for KORA [22]. For defining high triglycerides, we used a cut-off of 200 mg/dl [22]. For defining the hyperglycemia component, we used a cut-off of 140 mg/dl suggested by the American diabetes association diabetes diagnosis guideline to diagnose impaired glucose tolerance [23] or intake of antidiabetic medication.

### Statistical analysis

SOMAscan data was log2 transformed and each protein was standardized to have a mean of zero and a SD of 1 by subtracting its mean and dividing by its standard deviation to allow easier interpretation of the results per SD of log-transformed protein level.

Baseline characteristics were compared between the two cohorts using t-tests for continuous variables and chi-square tests with continuity correction for categorical variables.

### Proteome-wide analysis

Proteome-wide analyses to test for associations between prevalent and incident MetS and proteins were carried out using logistic regression with one model per protein. Each model had prevalent or incident MetS as the outcome, the log-transformed protein level as the explanatory variable, and was adjusted for age, sex, smoking status (categorized as never smoker, former smoker and current smoker) and physical activity (categorized as active vs inactive). We applied the Bonferroni method to correct for multiple testing throughout the paper. For the proteome-wide analyses this resulted in a significance threshold of p < 4.6e−05 (0.05/1095).

To replicate our results for prevalent MetS, we applied the same model in HUNT. We considered results replicated if they had consistent effect direction and survived Bonferroni correction calculated based on the number of KORA significant proteins.

Furthermore, we assessed the association of individual prevalent and incident MetS components with replicated prevalent MetS proteins and KORA incident MetS significant results, respectively. For incident components, analysis was done after removing participants with MetS at baseline. For each component (increased waist circumference, hypertriglyceridemia, reduced HDL, hyperglycemia and increased blood pressure), we applied the same model with the component as an outcome using the KORA data.

### Biomarker discovery for MetS

We investigated the predictive utility of the proteins significantly associated with incident MetS in KORA by utilizing the bootstrap ranking algorithm of the least absolute shrinkage and selection operator (LASSO) for model selection using the “elasso” R-package version 1.1 [24]. LASSO attempts to shrink the coefficients of the model covariates to zero thus selecting the covariates with the best predictive ability. We applied cross-validation to select the best LASSO constraint “lambda” within each bootstrap iteration.

We then used the area under the receiver operating characteristic curve (ROC-AUC) to test model performance calculated using the “pROC” R-package version 1.16.2 [25]. We further assessed performance using the calibration plot, which examines the agreement between observed and fitted values of the outcome [26] and by comparing the performance of LASSO selected protein model to a baseline model based on age and sex utilizing the DeLong test [27].

Additionally, we tested the performance of proteins associated with prevalent MetS as a biomarker panel using KORA as a training dataset and HUNT as a test dataset (full details in Additional file 1).

### Enrichment and protein–protein interaction network analyses

We used STRING [28] to evaluate the protein–protein interaction network of the MetS associated proteins (full details in Additional file 1).

### Mendelian randomization analysis

We used two-sample MR to investigate potential causal effects of replicated proteins on MetS. Mendelian randomization analysis is an instrumental variable (IV) analysis, in which genetic associations are used as anchors to assess causal effects of an exposure of interest on an outcome of interest. Two-sample MR entails the use of published genetic; i.e. single nucleotide polymorphism (SNP) association results to obtain IVs, thus allowing the use of the available bigger sample sizes and meta-analyses of genome wide association studies (GWAS).

First, we extracted SNPs associated with the protein of interest from already published genetic association studies using data of European ancestry. We extracted the IVs from SOMAscan GWAS studies by Suhre et al. (n = 1000) [19] and Sun BB et al. (n = 3301) [29] and the cis only association study by Emilsson et al. (n = 5457) [30].

We then identified ambiguous palindromic SNPs, which are SNPs with A/T or G/C alleles and an effect allele frequency around 0.5, using the cut-off points defined by the “TwoSampleMR” R-package [15]. We replaced the SNPs in question with an available proxy, defined as a SNP with r2 exceeding 0.85, or excluded them from further analyses [31]. To obtain a list of independent SNPs to be used as IVs in further analyses, we clumped the list of SNPs using the r2 cut-off of 0.001. Selected IVs had to be in cis with the protein of interest, i.e., within one Mb of the protein-coding gene as per the Human Genome Assembly GRCh37.p13. We subsequently extracted the outcome summary statistics of the selected IVs or of one of their proxies from the MetS GWAS study by Lind (n = 291,107) [11].

We used the Wald ratio to estimate a causal effect if there was only one IV available [32]. In cases where more IVs were available, we applied a random effects model of the inverse variance weighted meta-analysis to combine the Wald ratio estimates of all IVs [32, 33]. Whenever there was more than one IV, we ran the MR‐Egger regression model to check for horizontal pleiotropy in our causal models [34], and we investigated scatter plots, leave-one-out analysis plots and forest plots to identify outliers among the IVs that could be driving the results in a certain direction.

All analyses were done in R version 4.0.2 (The R Foundation for Statistical Computing). For MR analysis, the “TwoSampleMR” R-package version 0.5.5 was employed [15].

## Results

### Descriptive statistics of the study populations

Table 1 shows the baseline characteristics of both cohorts. The KORA sample comprised 998 participants with an age range of 43–75 years, of whom 515 were women, 371 had MetS at baseline and 147 developed it between baseline and follow-up. The HUNT sample compromised 923 participants with an age range of 31.6–91.7 years, of whom 235 were women and 418 had MetS. KORA participants had significantly lower waist circumference and triglyceride levels, and higher HDL levels, and were less often current smokers. Baseline characteristics of the follow-up subset of KORA used in incident MetS analyses are shown in Table 1.

**Table 1:** Baseline characteristics of the study populations

Variable	Prevalent MetS			Incident MetS
	KORA (n = 998)	HUNT (n = 923)	p value*	KORA (n = 623)
Age^a^ (years)	59.3 (43–75)	68.93 (31.6–91.7)	< 0.001	58.15 (43–74)
Sex female^b^	515 (51.6%)	235 (25.5%)	< 0.001	379 (60.8%)
BMI^c^ (kg/m^2^)	27.77 (4.58)	28.39 (3.97)	0.002	26.21 (3.87)
Waist circumference^c^ (cm)	94.56 (14.05)	100.18 (11.04)	< 0.001	89.07 (11.44)
Waist hip ratio^c^	0.89 (0.08)	0.96 (0.07)	< 0.001	0.86 (0.08)
Physically active^b^	620 (62.1%)	468 (50.7%)	< 0.001	416 (66.8%)
Smoking^b^			< 0.001	
Never smoker	423 (42.4%)	231 (25%)		277 (44.5%)
Former smoker	427 (42.8%)	497 (53.8%)		244 (39.2%)
Current smoker	148 (14.8%)	195 (21.1%)		102 (16.4%)
Total cholesterol^c^ (mg/dl)	221.99 (38.47)	178.50 (42.34)	NA**	222.83 (37.11)
HDL- cholesterol^c^ (mg/dl)	57.35 (15.19)	45.05 (11.25)	NA**	62.84 (14.45)
Triglyceride level^c^ (mg/dl)	129.06 (87.68)	161.82 (86.81)	NA**	96.59 (46.08)
Hypertension^d^	398 (39.9%)	382 (41.4%)	0.544	143 (23.0%)

^*^Continuous variables were tested for a difference between the two populations using t-tests and categorical variables with chi-square tests with continuity correction, **Differences between cohorts could not be statistically tested as KORA was measured in fasting samples and HUNT in non-fasting samples

^a^Mean (range)

^b^Number (percentage)

^c^Mean ± standard deviation. Hypertension was defined as having systolic blood pressure ≥ 140 mmHg and diastolic ≥ 90 mmHg or known medication-controlled hypertension. In HUNT we additionally used the ICD-10 codes I10–I15 of the hospital and primary care data and the codes K86 or K87 of the International Classification of Primary Care, Second Edition, to identify participants with hypertension

### Association results of plasma proteins with prevalent MetS

**Fig. 1:**
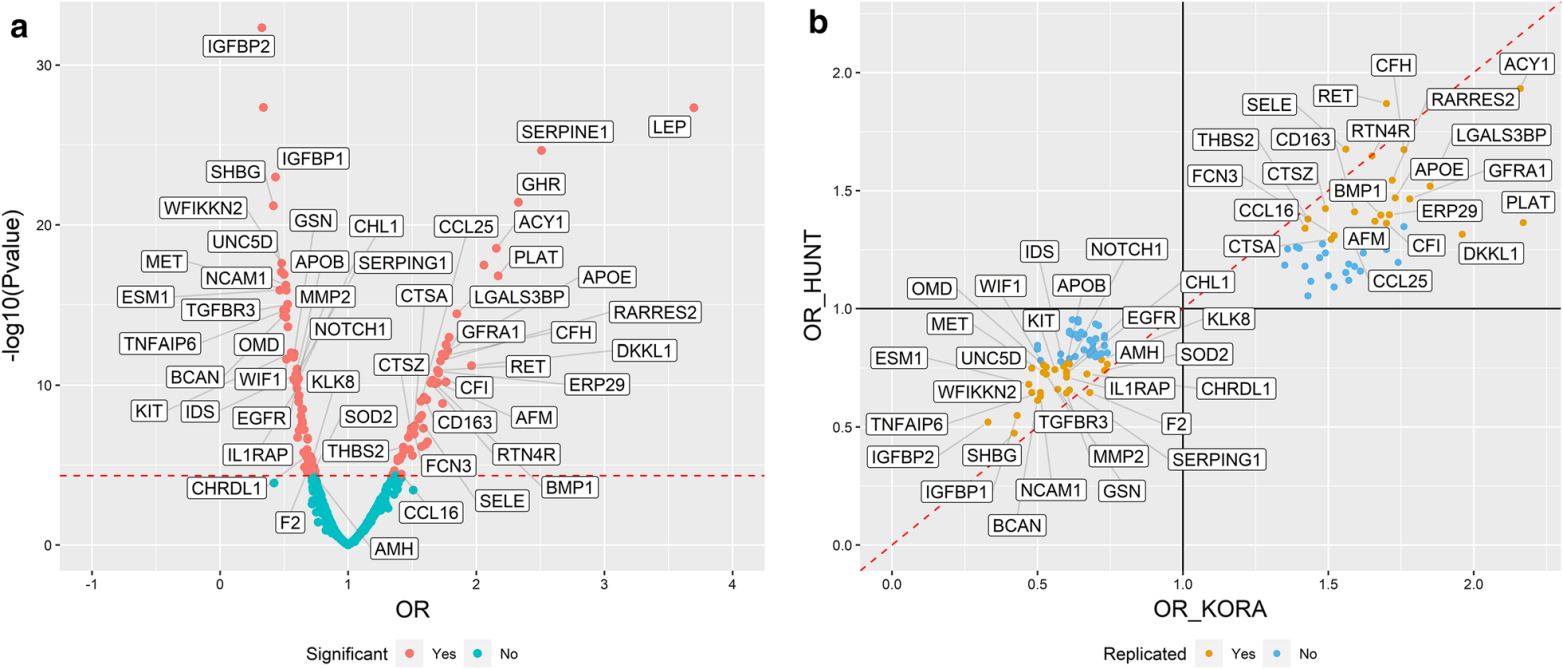
Results of proteome-wide analysis of prevalent MetS, with replicated proteins labelled by their gene name. **a** Volcano plot of the results in KORA. **b** Concordance plot examining effect sizes in KORA and HUNT. OR; odds ratio per 1 SD increase in log-transformed protein levels

The proteome-wide analysis of prevalent MetS yielded 116 Bonferroni significant proteins, of which 51 are positively associated with MetS and 65 are negatively associated (Additional file 2: Table S1). Of these, 53 successfully replicated in HUNT (Table 2; Fig. 1a). All of the 56 non-replicated proteins available in HUNT showed concordant direction of effect between the cohorts, and 35 of them were nominally significant in HUNT (Fig. 1b).

**Table 2:** Replicated results of the proteome-wide analysis of prevalent MetS in KORA and HUNT, sorted by the magnitude of the OR in KORA

Protein full name	UniProt	Gene symbol	KORA (n = 998)		HUNT (n = 923)	
			OR (95% CI)	P-value	OR (95% CI)	P-value
Leptin	P41159	LEP	3.70 (2.95–4.70)	4.77E-28	1.76 (1.49–2.08)	3.76E−11
Plasminogen activator inhibitor 1	P05121	SERPINE1	2.51 (2.12–3.00)	2.24E−25	1.37 (1.19–1.57)	9.99E−06
Growth hormone receptor	P10912	GHR	2.33 (1.97–2.78)	3.84E−22	2.08 (1.76–2.47)	9.44E−18
Tissue-type plasminogen activator	P00750	PLAT	2.17 (1.82–2.61)	1.54E−17	1.36 (1.19–1.57)	8.57E−06
Aminoacylase-1	Q03154	ACY1	2.16 (1.83–2.56)	2.95E−19	1.93 (1.64–2.30)	2.71E−14
Dickkopf-like protein 1	Q9UK85	DKKL1	1.96 (1.63–2.39)	6.02E−12	1.31 (1.15–1.51)	9.54E−05
Galectin-3-binding protein	Q08380	LGALS3BP	1.85 (1.59–2.16)	3.66E−15	1.52 (1.32–1.75)	6.81E−09
GDNF family receptor alpha-1	P56159	GFRA1	1.78 (1.52–2.09)	7.06E−13	1.46 (1.27–1.70)	1.99E−07
Complement factor H	P08603	CFH	1.76 (1.51–2.06)	1.44E−12	1.67 (1.42–1.98)	7.71E−10
Apolipoprotein E (isoform E3)	P02649	APOE	1.73 (1.49–2.02)	1.17E−12	1.47 (1.26–1.72)	1.16E−06
Retinoic acid receptor responder protein 2	Q99969	RARRES2	1.72 (1.48–2.01)	3.04E−12	1.54 (1.33–1.80)	1.84E−08
Endoplasmic reticulum resident protein 29	P30040	ERP29	1.71 (1.46–2.00)	1.39E−11	1.40 (1.21–1.61)	3.97E−06
Complement factor I	P05156	CFI	1.70 (1.46–2.00)	1.81E−11	1.36 (1.18–1.58)	2.72E−05
Proto-oncogene tyrosine-protein kinase receptor	P07949	RET	1.70 (1.46–1.99)	1.19E−11	1.87 (1.59–2.21)	1.71E−13
Bone morphogenetic protein 1	P13497	BMP1	1.68 (1.44–1.97)	8.25E−11	1.40 (1.17–1.68)	2.66E−04
Afamin	P43652	AFM	1.66 (1.43–1.93)	4.89E−11	1.37 (1.19–1.58)	1.28E−05
Reticulon-4 receptor	Q9BZR6	RTN4R	1.65 (1.42–1.92)	7.45E−11	1.65 (1.42–1.92)	6.57E−11
Scavenger receptor cysteine-rich type 1 protein M130	Q86VB7	CD163	1.59 (1.38–1.85)	5.75E−10	1.41 (1.23–1.62)	8.61E−07
C–C motif chemokine 25	O15444	CCL25	1.57 (1.36–1.82)	1.04E−09	1.33 (1.15–1.53)	7.50E−05
E-selectin	P16581	SELE	1.56 (1.34–1.82)	1.29E−08	1.68 (1.45–1.94)	3.29E−12
Ficolin-3	O75636	FCN3	1.52 (1.30–1.77)	1.15E−07	1.31 (1.13–1.52)	3.72E−04
Lysosomal protective protein	P10619	CTSA	1.51 (1.31–1.76)	4.55E−08	1.29 (1.12–1.49)	4.00E−04
Cathepsin Z	Q9UBR2	CTSZ	1.49 (1.29–1.73)	9.24E−08	1.42 (1.24–1.65)	1.35E−06
Thrombospondin-2	P35442	THBS2	1.43 (1.24–1.65)	7.57E−07	1.38 (1.20–1.60)	1.33E−05
C–C motif chemokine 16	O15467	CCL16	1.42 (1.21–1.68)	3.69E−05	1.34 (1.16–1.55)	5.95E−05
Insulin-like growth factor-binding protein 2	P18065	IGFBP2	0.33 (0.27–0.39)	4.63E−33	0.52 (0.44–0.62)	4.04E−14
Sex hormone-binding globulin	P04278	SHBG	0.42 (0.35–0.50)	6.47E−22	0.47 (0.40–0.56)	2.86E−18
Insulin-like growth factor-binding protein 1	P08833	IGFBP1	0.43 (0.37–0.51)	1.03E−23	0.55 (0.47–0.64)	1.98E−14
Endothelial cell-specific molecule 1	Q9NQ30	ESM1	0.47 (0.39–0.56)	1.20E−16	0.68 (0.57–0.80)	1.31E−05
Netrin receptor UNC5D	Q6UXZ4	UNC5D	0.48 (0.40–0.56)	8.72E−18	0.75 (0.64–0.87)	1.92E−04
WAP, Kazal, immunoglobulin, Kunitz and NTR domain-containing protein 2	Q8TEU8	WFIKKN2	0.48 (0.41–0.57)	2.52E−18	0.65 (0.56–0.75)	3.52E−09
Brevican core protein	Q96GW7	BCAN	0.50 (0.42–0.59)	4.72E−15	0.61 (0.51–0.73)	1.87E−07
Neural cell adhesion molecule 1, 120 kDa isoform	P13591	NCAM1	0.51 (0.44–0.60)	6.01E−17	0.63 (0.54–0.73)	1.10E−09
Tumor necrosis factor-inducible gene 6 protein	P98066	TNFAIP6	0.51 (0.44–0.60)	2.05E−15	0.65 (0.56–0.75)	5.30E−09
Wnt inhibitory factor 1	Q9Y5W5	WIF1	0.52 (0.43–0.62)	2.39E−12	0.76 (0.66–0.88)	1.62E−04
Hepatocyte growth factor receptor	P08581	MET	0.52 (0.44–0.60)	1.22E−16	0.73 (0.63–0.85)	4.76E−05
Osteomodulin	Q99983	OMD	0.53 (0.45–0.62)	2.38E−14	0.75 (0.65–0.87)	1.21E−04
Transforming growth factor beta receptor type 3	Q03167	TGFBR3	0.53 (0.45–0.62)	9.27E−16	0.72 (0.62–0.84)	1.78E−05
Mast/stem cell growth factor receptor Kit	P10721	KIT	0.56 (0.47–0.65)	9.54E−13	0.74 (0.64–0.86)	5.65E−05
Gelsolin	P06396	GSN	0.57 (0.49–0.67)	1.83E−12	0.66 (0.57–0.76)	3.50E−08
Iduronate 2-sulfatase	P22304	IDS	0.59 (0.51–0.69)	2.21E−11	0.76 (0.65–0.87)	1.70E−04
72 kDa type IV collagenase	P08253	MMP2	0.60 (0.51–0.69)	2.09E−11	0.64 (0.56–0.74)	3.08E−09
Neural cell adhesion molecule L1-like protein	O00533	CHL1	0.60 (0.51–0.69)	2.41E−11	0.73 (0.63–0.83)	6.26E−06
Epidermal growth factor receptor	P00533	EGFR	0.60 (0.51–0.70)	7.17E−11	0.73 (0.62–0.86)	2.28E−04
Interleukin-1 Receptor accessory protein	Q9NPH3	IL1RAP	0.60 (0.51–0.70)	1.61E−10	0.71 (0.62–0.82)	1.61E−06
Apolipoprotein B	P04114	APOB	0.60 (0.52–0.69)	9.93E−12	0.77 (0.67–0.88)	1.66E−04
Neurogenic locus notch homolog protein 1	P46531	NOTCH1	0.61 (0.52–0.70)	4.64E−11	0.77 (0.67–0.88)	1.63E−04
Plasma protease C1 inhibitor	P05155	SERPING1	0.61 (0.53–0.71)	3.89E−11	0.66 (0.56–0.77)	1.80E−07
Chordin-like protein 1	Q9BU40	CHRDL1	0.67 (0.57–0.79)	2.86E−06	0.72 (0.60–0.86)	4.50E−04
Thrombin	P00734	F2	0.68 (0.57–0.81)	2.65E−05	0.65 (0.55–0.75)	4.13E−08
Kallikrein-8	O60259	KLK8	0.72 (0.62–0.83)	5.94E−06	0.79 (0.69–0.90)	4.12E−04
Superoxide dismutase [Mn], mitochondrial	P04179	SOD2	0.73 (0.63–0.84)	2.42E−05	0.74 (0.64–0.85)	3.13E−05
Muellerian-inhibiting factor	P03971	AMH	0.74 (0.64–0.85)	4.28E−05	0.77 (0.66–0.88)	4.05E−04

All analyses were adjusted for age, sex, smoking status and physical activity

OR; odds ratio per 1 SD increase in log-transformed protein levels

Among the replicated proteins, insulin-like growth factor-binding protein 2 (IGFBP2) had the lowest odds ratio (OR) in both cohorts per SD increase in log-transformed protein level, with values of 0.33 (95% CI 0.27–0.39) in KORA and of 0.52 (95% CI 0.44–0.62) in HUNT; and leptin had the highest OR in both cohorts, with values of 3.7 (95% CI 2.95–4.7) in KORA and 1.76 (95% CI 1.49–2.08) in HUNT. The correlation matrices of replicated proteins are shown in Additional file 1: Figure S1.

### Association results of plasma proteins with incident MetS

**Fig. 2:**
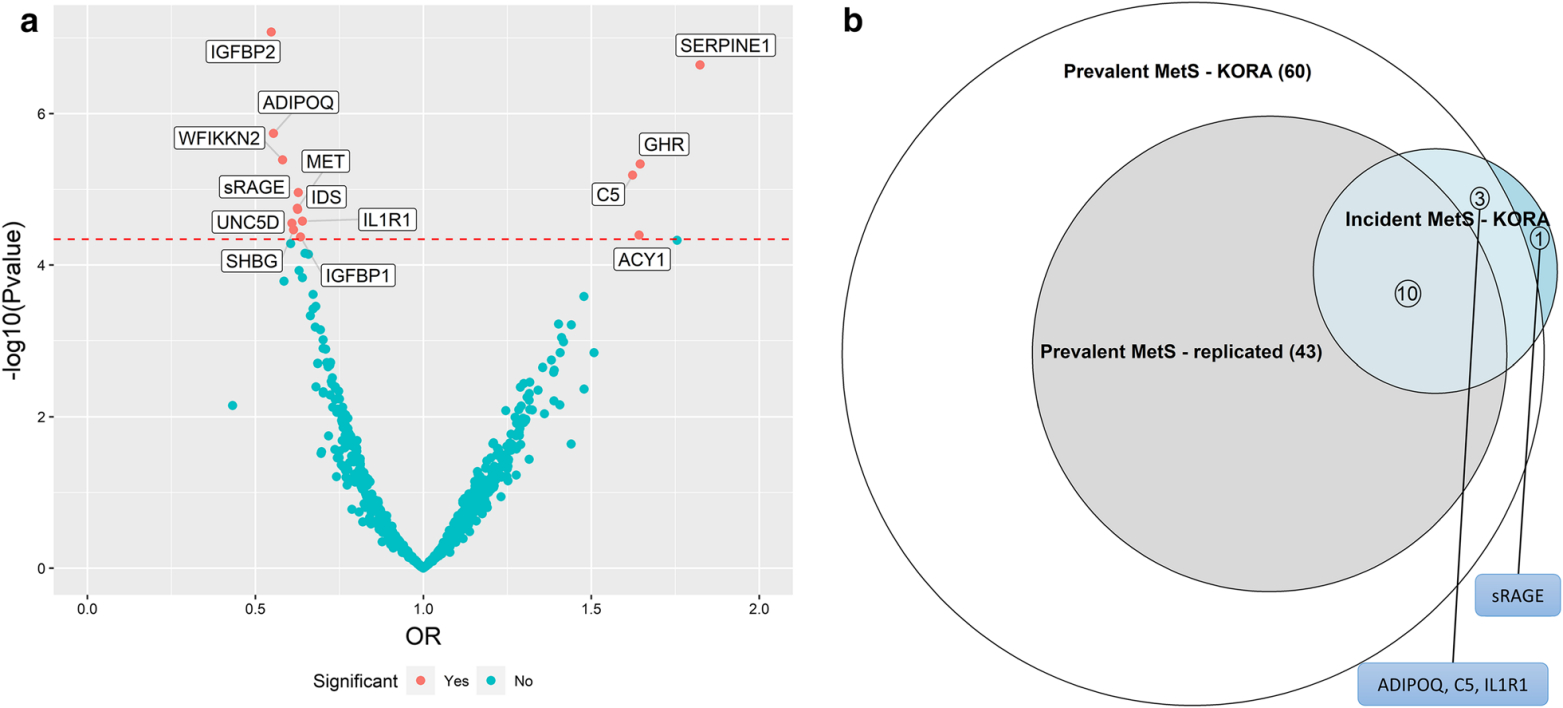
Results of proteome-wide analysis of incident MetS in KORA. **a** Volcano plot with Bonferroni significant proteins labelled by their gene name. **b** Euler diagram showing extent of overlap between incident MetS results in KORA and prevalent MetS results in KORA and its replicated results in HUNT. OR; odds ratio per 1 SD increase in log-transformed protein levels

The proteome-wide analysis of incident MetS in KORA yielded 14 significant protein associations at a Bonferroni corrected threshold (Table 3; Fig. 2). IGFBP2 was the most strongly associated protein based on p-value (OR = 0.55; 95% CI = 0.44–0.68) and plasminogen activator inhibitor 1 (SERPINE1) had the largest magnitude of association (OR = 3.70; 95% CI = 2.95–4.70). The incident MetS significant proteins included 10 overlapping the replicated results and 13 overlapping the KORA significant results of prevalent MetS (Fig. 2b). Only soluble advanced glycosylation end product-specific receptor (sRAGE) (OR = 0. 63; 95% CI = 0.51–0.77) was unique to incident MetS.

**Table 3:** Bonferroni significant results of the proteome-wide analysis with incident MetS in KORA (N = 623), sorted by the magnitude of the OR

Target full name	UniProt	Gene symbol	OR (CI)	P-value
Plasminogen activator inhibitor 1	P05121	SERPINE1	1.82 (1.46–2.30)	2.28E−07
Growth hormone receptor	P10912	GHR	1.65 (1.33–2.04)	4.63E−06
Aminoacylase-1	Q03154	ACY1	1.64 (1.30–2.09)	4.02E−05
C5a anaphylatoxin	P01031	C5	1.62 (1.32–2.01)	6.52E−06
Adiponectin	Q15848	ADIPOQ	0.55 (0.43–0.70)	1.83E−06
Insulin-like growth factor-binding protein 2	P18065	IGFBP2	0.55 (0.44–0.68)	8.36E−08
WAP, Kazal, immunoglobulin, Kunitz and NTR domain-containing protein 2	Q8TEU8	WFIKKN2	0.58 (0.46–0.73)	4.09E−06
Netrin receptor UNC5D	Q6UXZ4	UNC5D	0.61 (0.48–0.77)	2.82E−05
Sex hormone-binding globulin	P04278	SHBG	0.61 (0.49–0.77)	3.43E−05
Iduronate 2-sulfatase	P22304	IDS	0.63 (0.50–0.77)	1.84E−05
Hepatocyte growth factor receptor	P08581	MET	0.63 (0.50–0.77)	1.78E−05
Advanced glycosylation end product-specific receptor, soluble	Q15109	AGER	0.63 (0.51–0.77)	1.10E−05
Insulin-like growth factor-binding protein 1	P08833	IGFBP1	0.63 (0.51–0.79)	4.26E−05
Interleukin-1 receptor type 1	P14778	IL1R1	0.64 (0.52–0.79)	2.64E−05

All analyses were adjusted for age, sex, smoking status and physical activity

OR; odds ratio per 1 SD increase in log-transformed protein levels

### MetS components analysis

**Fig. 3:**
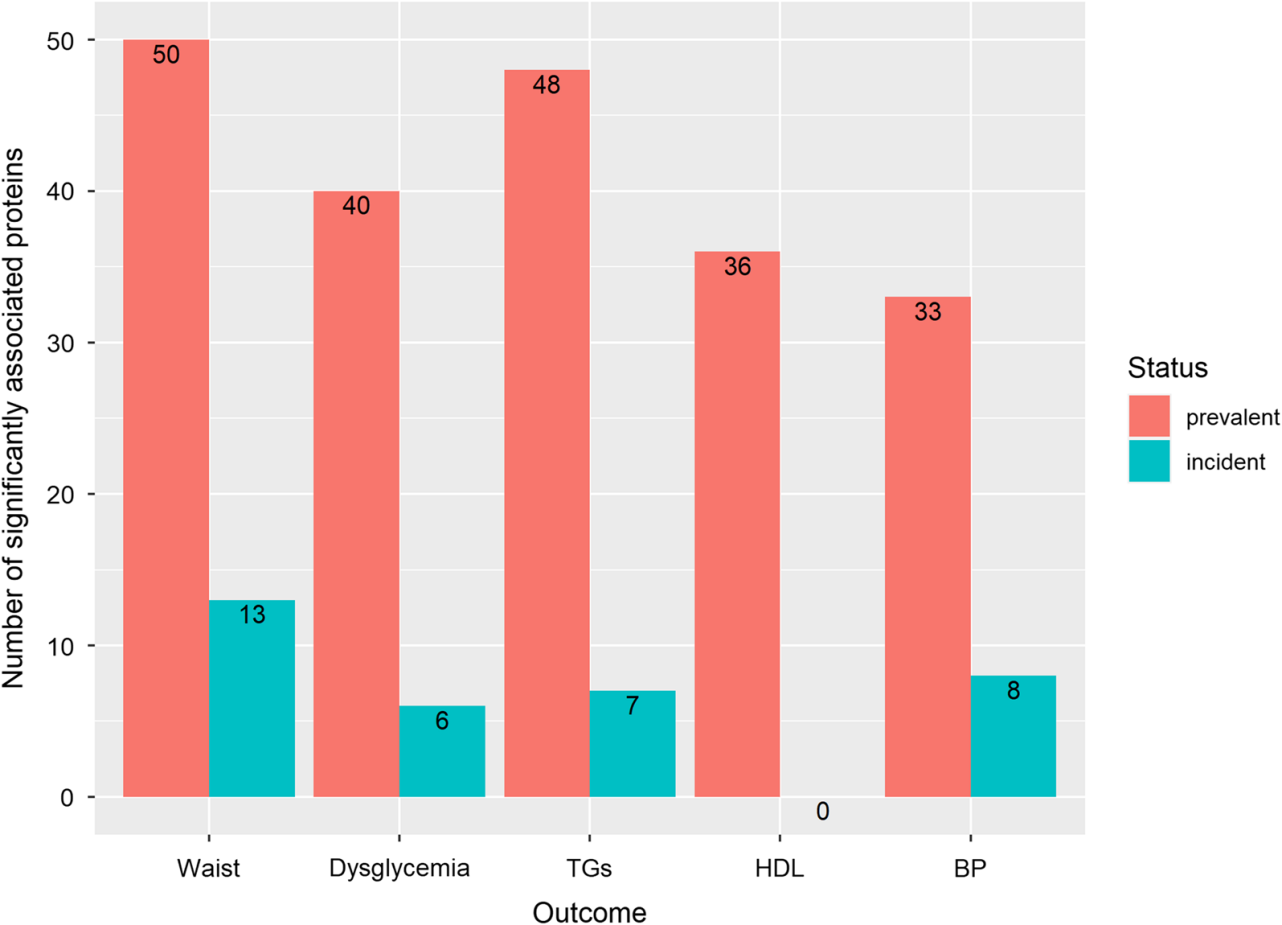
Barplot showing protein associations of prevalent and incident individual MetS components with replicated prevalent MetS results (n = 53 proteins) and KORA incident MetS results (n = 14 proteins) respectively. The abbreviations used in the y-axis for the MetS components are: Waist (increased waist circumference); dysglycemia (increased blood glucose level); TGs (hypertriglyceridemia); HDL (reduced HDL); BP (increased blood pressure)

For each prevalent component, we tested whether the prevalent-MetS-replicated proteins were also associated with the component. Each component was associated with at least 33 of these proteins, with increased waist circumference and hypertriglyceridemia showing the highest number of associations with 50 and 48, respectively (Fig. 3; Additional file 2: Table S2). In total, 18 proteins were common to all prevalent components (Additional file 2: Table S2).

For the incident components, increased waist circumference and high blood pressure were associated with 13 and 8 proteins out of the 14 incident MetS KORA protein associations respectively (Additional file 2: Table S3). ADIPOQ and IGFBP2 were associated with the four incident components with significant results namely increased waist circumference, hypertriglyceridemia, hyperglycemia and increased blood pressure.

### Biomarker discovery

We explored the utility of proteins associated with incident MetS as predictive biomarkers in KORA. The LASSO-selected predictive model included 8 proteins (Additional file 2: Table S4) and had an AUC of 0.75 (95% CI = 0.71–0.79). Comparing the LASSO selected predictive model to the age and sex model yielded a delta AUC of 0.12 in KORA, which was significant based on the DeLong test (Additional file 1: Figure S2). The top 2 performing protein were netrin receptor (UNC5D) with AUC = 0.66 (CI = 0.66–0.71) and aminoacylase-1 (ACY1) with AUC = 0.65 (CI = 0.60–0.70) (Additional file :2Table S5).

Our investigation in the utility of prevalent MetS protein associations as biomarkers yielded a 15-protein diagnostic model. The model yielded lower performance in HUNT with an AUC-KORA of 0.87 (95% CI = 0.85–0.89) and an AUC-HUNT of 0.74 (95% CI = 0.71–0.77) (Additional file 1).

### Mendelian randomization

**Fig. 4:**
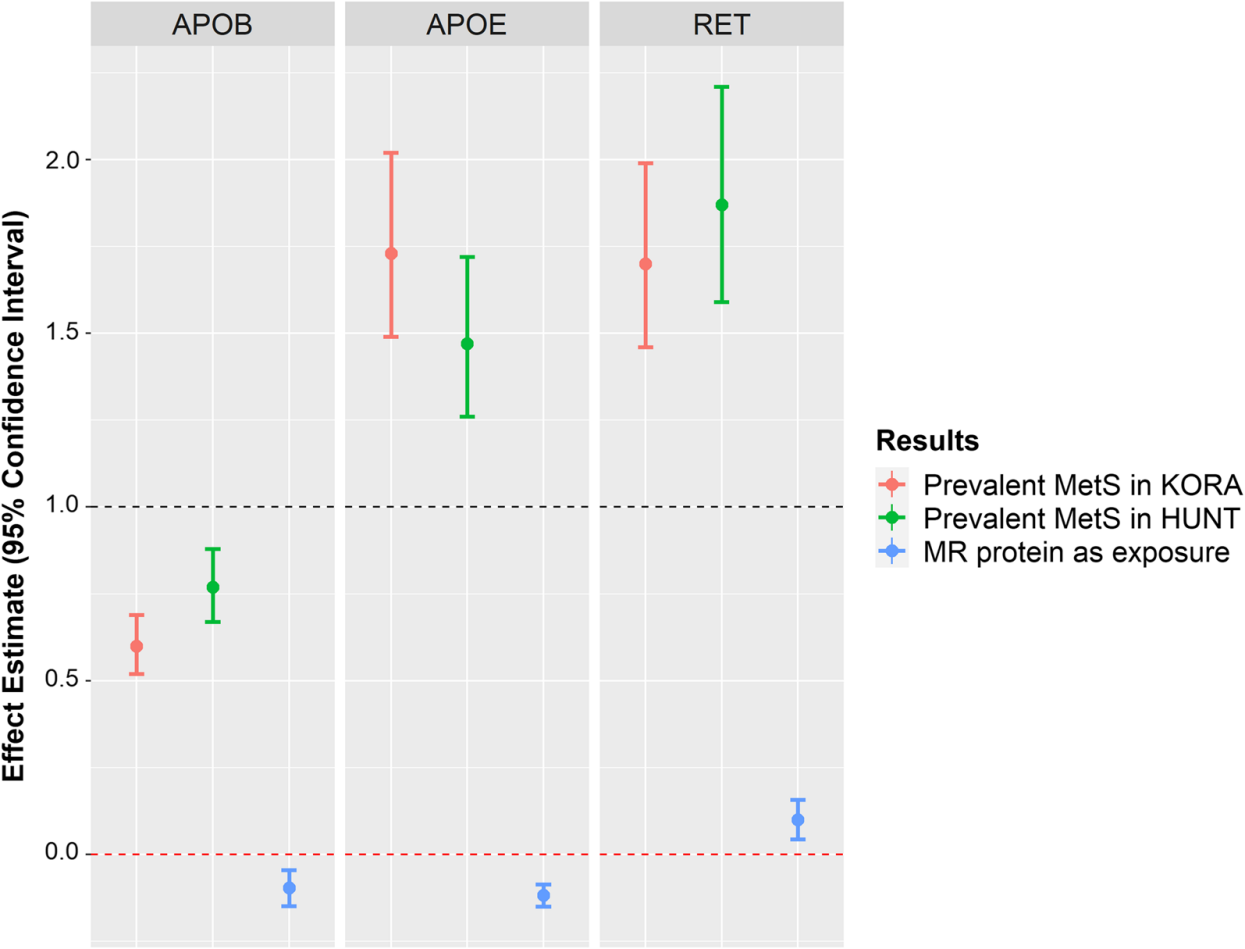
Mendelian randomization results with MetS as outcome compared with observational effect estimates. Effect estimates represent odds ratios for association results and represent beta coefficients for MR with proteins as exposure

We explored if the proteins were causal to MetS (Additional file 2: Table S6). Of the 29 proteins for which we found IVs, 3 showed Bonferroni significant causal effects on MetS (Fig. 4), namely apolipoprotein E3 (APOE3) (Wald-Ratio = − 0.12, Wald-p = 3.63e−13), apolipoprotein B (APOB) (Wald-Ratio = − 0.09, Wald-p = 2.54e−04) and proto-oncogene tyrosine-protein kinase receptor (RET) (Wald-Ratio = 0.10, Wald-p = 5.40e−04).

## Discussion

We used aptamer-based proteomics to investigate plasma protein associations with prevalent and incident MetS and, for those proteins showing a relationship with this syndrome, examined their utility as biomarkers and assessed their causal relationship with MetS. Of the 116 proteins associated with prevalent MetS in the KORA F4 study, 53 successfully replicated in the HUNT3 study. The proteins with the largest effect estimates were leptin and IGFBP2, both of which have been previously reported to be associated with obesity, T2D and MetS [35–38]. The replicated results also included 30 new protein associations, not previously reported to be associated with MetS, including neural cell adhesion molecule L1-like protein (CHL1), complement factor I (CFI), GDNF family receptor alpha-1 (GFRA1), kallikrein-8 (KLK8), brevican core protein (BCAN), dickkopf-like protein 1 (DKKL1), netrin receptor (UNC5D), NTR domain-containing protein 2 (WFIKKN2), and endoplasmic reticulum protein 29 (ERP29).

Replicated proteins overlap with the protein associations with body mass index (BMI) and type 2 diabetes. WFIKKN2, a protease inhibitor, was reported to be negatively associated with BMI with potential bi-directional causal effect as demonstrated by MR analysis [36]. ERP29, a chaperone protein, has been reported to be positively associated with BMI and to have a role in proinsulin secretion [39]. Of the replicated proteins, endothelial cell-specific molecule 1 (ESM1), has been reported to be low in liver steatosis in MetS patients [40] and in macro-albuminuria in T2D patients [41], both of which are in line with the negative association between ESM1 and MetS observed here. However, ESM1 was reported to be positively associated with atherosclerotic CVD [42].

Associations with incident MetS overlapped with prevalent MetS results, except for sRAGE, which was unique to incident MetS. sRAGE acts as a decoy of the RAGE cell surface receptor. sRAGE exogenously traps advanced glycation end products, therefore decreasing their harmful inflammatory effects through the blockage of their action on RAGE. sRAGE has been previously reported to be inversely associated with T2D, BMI and MetS [36, 43, 44] and was reported to lower the risk of CVD in T2D patients through the modulation of cardiovascular cell apoptosis [45]. RAGE-knockout mice were shown to suffer from accelerated weight gain, hypercholesterolemia and increased insulin levels pointing to the potential complex role of the RAGE family of receptors in the pathogenesis of insulin resistance and obesity [46].

To assess which of the MetS components are driving our observed results, we explored potential associations between our replicated protein associations with prevalent MetS and the respective MetS components and between incident MetS significant proteins and the respective MetS components. In total, 18 of the 53 replicated proteins were associated with all prevalent components. Of them, five were previously reported to be associated with all MetS components namely leptin, IGFBP1, IGFBP2, tissue-type plasminogen activator (PLAT) and SERPINE1 [12]. Ten of these 18 proteins (leptin, IGFBP1, IGFBP2, SHBG, growth hormone receptor (GHR), hepatocyte growth factor receptor (MET), galectin-3-binding protein (LG3BP), APOB and Wnt inhibitory factor 1 (WIF-1)) were previously reported to be associated with T2D, reflecting the shared pathogenic pathways between the two entities [47, 48]. Moreover, 4 of the 18 proteins (PLAT, SERPINE1, 72 kDa type IV collagenase (MMP2), NCAM1) have been reported to be associated with CVD, providing further evidence for the link between CVD and MetS. However, in the present study MMP2 showed a negative association with MetS, contradicting previous reports [49]. While MMP2 has been reported to be increased in metabolic syndrome and cardiovascular disease, its deficiency has also been reported to be associated with metabolic and inflammatory pathologies, pointing toward a complex relationship of MMP2 with cardiometabolic disorders [50, 51].

Of the incident MetS components, ADIPOQ and IGFBP2 were common to all incident components except for reduced HDL, which showed no protein associations. ADIPOQ and IGFBP2 were reported before to be associated with T2D and obesity [36, 52, 53].

Moreover, we evaluated the performance of the proteins as prediction biomarkers, both as a risk score and as individual biomarkers. As predictors of future MetS, the risk score had moderate performance in KORA (AUC = 0.75). As single predictive biomarkers the top five proteins included UNC5D, ACY1, SERPINE1, sRAGE and C5a anaphylatoxin. The lower performance of the proteins as biomarkers could be partly attributed to the differences in baseline characteristics of both cohorts and to the definition of the MetS, which relies on arbitrarily defined cut-off points based on risk assessment of its different components.

The investigation into the causal effects of proteins on MetS showed evidence for 2 protective casual proteins—APOE3 and APOB—and one harmful, RET. Except for APOE3, the causal effect of the proteins had the same effect direction as their observational results. APOE3 is an isoform of the APOE gene, which is a protein-coding gene with two other isoforms, namely APOE2 and APOE4. The APOE isoforms are encoded by two SNPs namely rs429358-C/T and rs7412-C/T. The combination of rs429358-T and rs7412-T is characteristic of the second isoform, of rs429358-T and rs7412-C is characteristic of the third isoform and of rs429358-C and rs7412-C is characteristic of the fourth isoform [54].

The discrepancy between MR and observational results of APOE3 could be due to the fact that the causal effect represents lifetime exposure in comparison to the observational time point effect. Additionally, the IV used in the MR analysis rs1065853 with the effect allele T, is in LD with the T allele of the SNP rs7412. The T allele of the SNP rs7412 characterizes the genotype of the APOE2 polymorphism, indicating that the MR result reflects the effect of APOE2 and not APOE3.APOE2 has been reported to be associated with lower risk of MetS in Uyghur ethnic men [55], with longevity [56] and with lower risk of Alzheimer’s disease.

### Strengths and limitations

Through the use of the high throughput aptamer based SOMAscan platform, we assessed the association of MetS with a large number of proteins (1095 in total). The use of plasma samples allowed us to find associations which may reflect the processes of multiple tissues and pathways that may be involved in the pathogenesis of MetS; as plasma is easily accessible, our discovered associations may be more readily transferable for use as clinical biomarkers. The replication in the HUNT study indicates broader generalizability of our results. The application of MR to decipher the causal framework governing these associations will enable future investigators to prioritize our results toward drug target identification and further functional investigation of MetS.

There are a number of limitations to our study. We were not able to apply the same MetS definition in the replication study HUNT as in the discovery KORA as the former lacked fasting blood sample collection; however, we used clinically defined cut-off points of non-fasting measurements that reflect the same pathologies identified using fasting measurements. Notably, a study comparing MetS-scores defined using fasting vs. non-fasting samples found that both scores were linked to the development of coronary artery disease and diabetes [57]. The aptamer-based technique could suffer from cross-reactivity; however, our results included proteins replicating previously reported associations measured using techniques other than SOMAscan [58]. The analysis of incident MetS were conducted in a smaller sample size than prevalent MetS and we could not investigate replication in HUNT due to the lack of follow-up data.

We applied rigorous methods in our causality analysis using MR to use only valid IVs and to apply sensitivity analyses to evaluate pleiotropy. However, MR is dependent on multiple assumptions that are hard to verify and test and its results should be interpreted with caution. Moreover, the studies we used in the causal analyses differed in power for the exposures and the outcome, with MetS GWAS having a bigger sample size and subsequently more power than the protein GWAS studies.

## Conclusion

Our results provide a comprehensive analysis of the associations between plasma proteins and MetS. Replicated results included proteins previously reported to be associated with cardio-metabolic traits, thus pointing to pathogenic pathways they share with MetS, including insulin resistance and CVD. These proteins include leptin, GHR, SHBG, IGFBP1 and IGFBP2. Replicated results also included proteins involved in the pathogenesis of CVD, such as PLAT, SERPINE1 and members of the complement system. Our replicated results identified new proteins including ERP29, KLK8, DKKL1 and WFIKKN2. We identify sRAGE to be uniquely associated with the incidence of MetS, which is in line with the observed phenotype in sRAGE knockout mice models.

Biomarker analysis identified an eight proteins predictive panel with an AUC of 0.75. Moreover, causal analysis using Mendelian randomization suggested causal effects of APOE2, APOB and RET on MetS. Further functional studies are needed to clarify their roles in the pathogenesis of MetS.

## Supplementary Information

**Additional file 1: Figure S1.** Pearson’s correlation plot of replicated proteins in: A) KORA; B) HUNT. **Figure S2.** ROC curve comparing the bootstrap ranking LASSO selected protein model with age and sex model predicting incident MetS in KORA, showing the AUCs, their 95% CI and the difference (delta AUC) and p-value of the DeLong test comparing both models. **Figure S3.** ROC curve comparing the bootstrap ranking LASSO selected protein model with age and sex model predicting prevalent MetS in KORA (A) and HUNT (B), showing the AUCs, their 95% CI and the difference (delta AUC) and p-value of the DeLong test comparing both models. **Figure S4.** Calibration plots of the bootstrap ranking LASSO-selected MetS diagnostic model in: A) KORA; B) HUNT. **Figure S5.** STRING protein-protein interaction network constructed using the prevalent or incident MetS associated proteins in KORA without adding additional interactor proteins.

**Additional file 2:** **Table S1.** Proteins significantly associated with prevalent MetS in KORA, their corresponding results in HUNT as well as their random effect meta-analysis results. **Table S2.** Overlap between the results of prevalent MetS components and replicated prevalent MetS results. **Table S3.** Overlap between the results of incident MetS components and incident MetS KORA results. **Table S4.** Coefficients of the incident MetS predictive protein risk score. **Table S5.** Results of the 14 significantly associated proteins with incident MetS as single predictive biomarkers in KORA. **Table S6.** Results of Mendelian randomization analysis with proteins as the exposure and MetS as the outcome. **Table S7.** Coefficients of the prevalent MetS diagnostic protein risk score. **Table S8.** Results of the 116 significantly associated proteins with prevalent MetS as single diagnostic biomarkers in KORA and HUNT.

## Data Availability

The KORA data is available through application at the KORA Project Application Self-Service Tool (https://epi.helmholtz-muenchen.de/). The HUNT data is available through application to the HUNT Research Centre (http://www.ntnu.edu/hunt/data). Data used in the two-sample MR analysis are publicly available and can be accessed through: MetS GWAS by Lind using the GWAS Catalog accession (GCST009602); Sun et al. at: http://www.phpc.cam.ac.uk/ceu/proteins/; Suhre et al. at: http://proteomics.gwas.eu; and Emilsson et al. at www.sciencemag.org/content/361/6404/769/suppl/DC1.

## Abbreviations

MetS: Metabolic syndrome
T2D: Type 2 diabetes
CVD: Cardiovascular diseases
CAD: Coronary artery disease
MR: Mendelian randomization
KORA: Cooperative health research in the Region of Augsburg
HUNT: The Nord-Trøndelag Health Study
HDL: High density lipoprotein cholesterol
LASSO: Least absolute shrinkage and selection operator
ROC-AUC: Area under the receiver operating characteristic curve
SNP: Single nucleotide polymorphism
GWAS: Genome wide association studies
BMI: Body mass index
