# Supplementary material for: Metabolic syndrome and the plasma proteome: from association to causation
Source: Cardiovasc Diabetol. 2021 May 20;20:111. doi: 10.1186/s12933-021-01299-2 (PMC8138979; doi:10.1186/s12933-021-01299-2)
Supplement: Supplementary file 1 — Additional file 1: Figure S1. Pearson’s correlation plot of replicated proteins in: A) KORA; B) HUNT. Figure S2. ROC curve comparing the bootstrap ranking LASSO selected protein model with age and sex model predicting incident MetS in KORA, showing the AUCs, their 95% CI and the difference (delta AUC) and p-value of the DeLong test comparing both models. Figure S3. ROC curve comparing the bootstrap ranking LASSO selected protein model with age and sex model predicting prevalent MetS in KORA (A) and HUNT (B), showing the AUCs, their 95% CI and the difference (delta AUC) and p-value of the DeLong test comparing both models. Figure S4. Calibration plots of the bootstrap ranking LASSO-selected MetS diagnostic model in: A) KORA; B) HUNT. Figure S5. STRING protein-protein interaction network constructed using the prevalent or incident MetS associated proteins in KORA without adding additional interactor proteins. [file 12933_2021_1299_MOESM1_ESM.docx]

**Proteins as biomarkers of prevalent MetS**

We applied the same model selection strategy described in the methods section to build a protein risk score of prevalent MetS. We used KORA as a training dataset and HUNT as a test dataset to identify the subset of proteins with the highest predictive abilities.

We investigated the utility of the proteins significantly associated with prevalent MetS in KORA and available in HUNT (109 proteins) as biomarkers. We applied bootstrap ranking of LASSO to select the proteins with the best performance, resulting in a 15-protein diagnostic model (Supplementary Table S7). The LASSO selected model performed well in both KORA and HUNT, with an AUC-KORA of 0.87 (95% CI = 0.85 - 0.89) and AUC-HUNT of 0.74 (95% CI = 0.71 - 0.77) (Supplementary Figure S3).

Comparing the LASSO selected diagnostic model to the age and sex model yielded an increase in AUC of 0.19 in KORA and 0.24 in HUNT, both of which were significant based on the DeLong test (Supplementary Figure S3).

Investigation of the LASSO-selected model using calibration plots, examining fitted vs observed values of the outcome, yielded a calibration-in-the-large (intercept) of 0.56 and a calibration slope of 0.55 in HUNT (Supplementary Figure S4). Calibration-in-the-large is optimally zero, which would indicate no difference between observed and model-predicted risks. In HUNT, we obtained values higher than zero, indicating higher observed cases than predicted. The calibration slope in HUNT was 0.53, which indicates possible overfitting of the model and the need for coefficient shrinkage.

As single biomarkers, the top performing protein was adiponectin with an AUC = 0.75 (95% CI = 0.72–0.78) (Supplementary table S8). The highest performing proteins overlapping both cohorts, based on their average AUC, were SHBG with KORA-AUC = 0.72 (95% CI = 0.69–0.75) and HUNT-AUC = 0.66 (95% CI = 0.63–0.70) and insulin-like growth factor-binding protein 1 (IGFBP1) with KORA-AUC = 0.73 (95% CI = 0.69–0.76) and HUNT-AUC = 0.65 (95%CI = 0.62–0.69).

The diagnostic risk score performed well in KORA; however, the performance was poorer in HUNT. Further investigation using calibration plots revealed that this decrease in performance could be driven by the differences in MetS definition between the cohorts. The calibration slope in HUNT indicated the need for coefficient shrinkage attributable to the heterogeneity between the cohorts in terms of patient characteristics and outcome definition. As single diagnostic biomarkers, the top five performers included adiponectin, PLAT, IGFBP1, IGFBP2, and SHBG.

**Enrichment and protein-protein interaction network analyses**

Using STRING (1), we investigated a protein-protein interaction network and ran enrichment analysis. To crease the network, we used the 113 unique gene identifiers of the proteins associated with prevalent or incident MetS in KORA while specifying that no other protein interactors be added to the network by STRING. We used the SOMAscan measured protein genes as background. Supplementary Figure S5 shows the protein-protein interaction network. The network interaction analysis was significant with 113 nodes and 397 edges, with an expected number of edges of 320, resulting in an enrichment p-value of 1.72e-05. Functional enrichment of the network yielded two significant results: enriched cellular components: extracellular region (p=1.57e-05) and extracellular space (p=0.0029). Five UniProt annotated keywords were also significant, namely Signal, Secreted, Disulfide bond, Glycoprotein and Repeat.


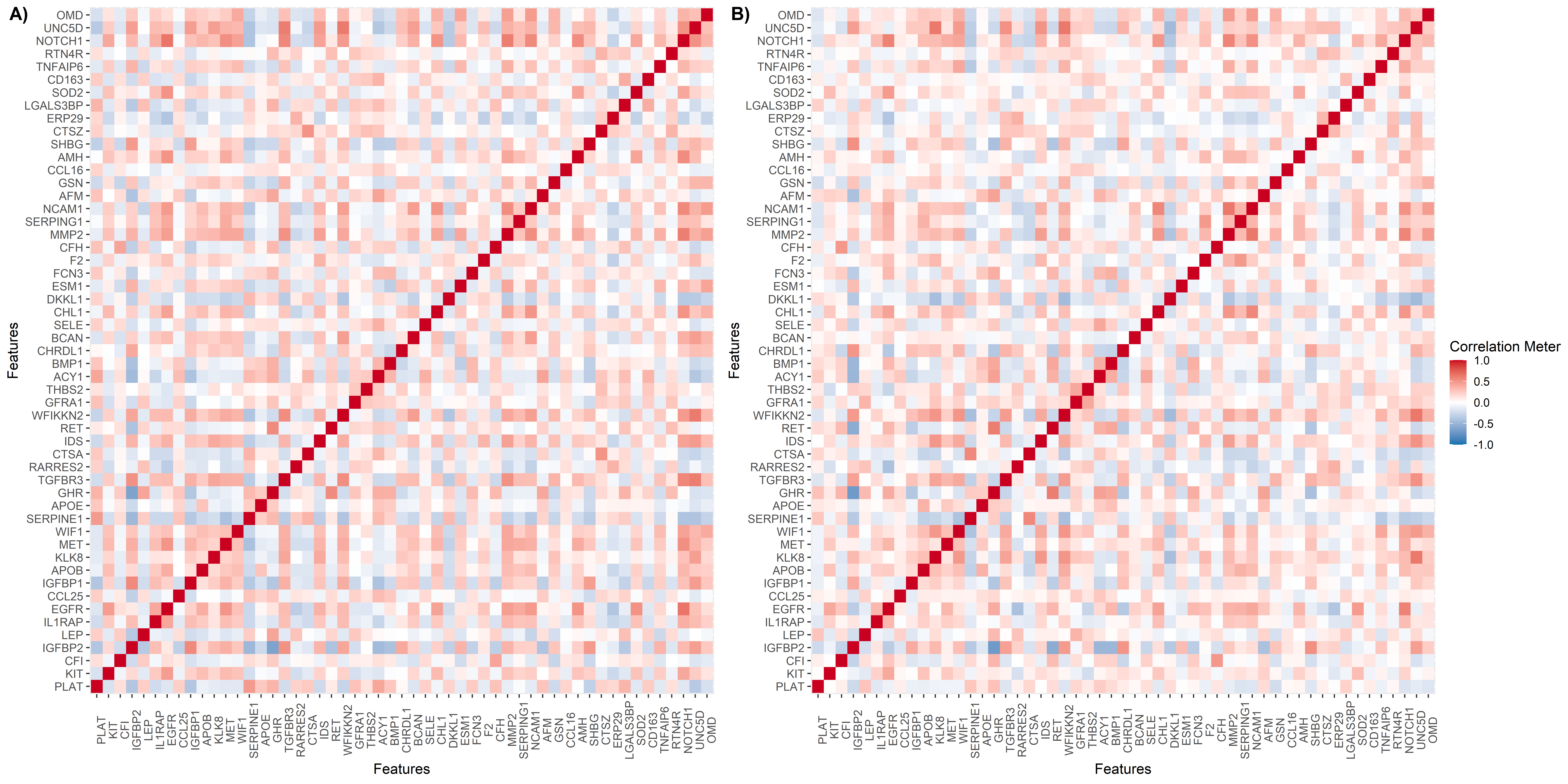


Figure S1: Pearson’s correlation plot of replicated proteins in: A) KORA; B) HUNT.


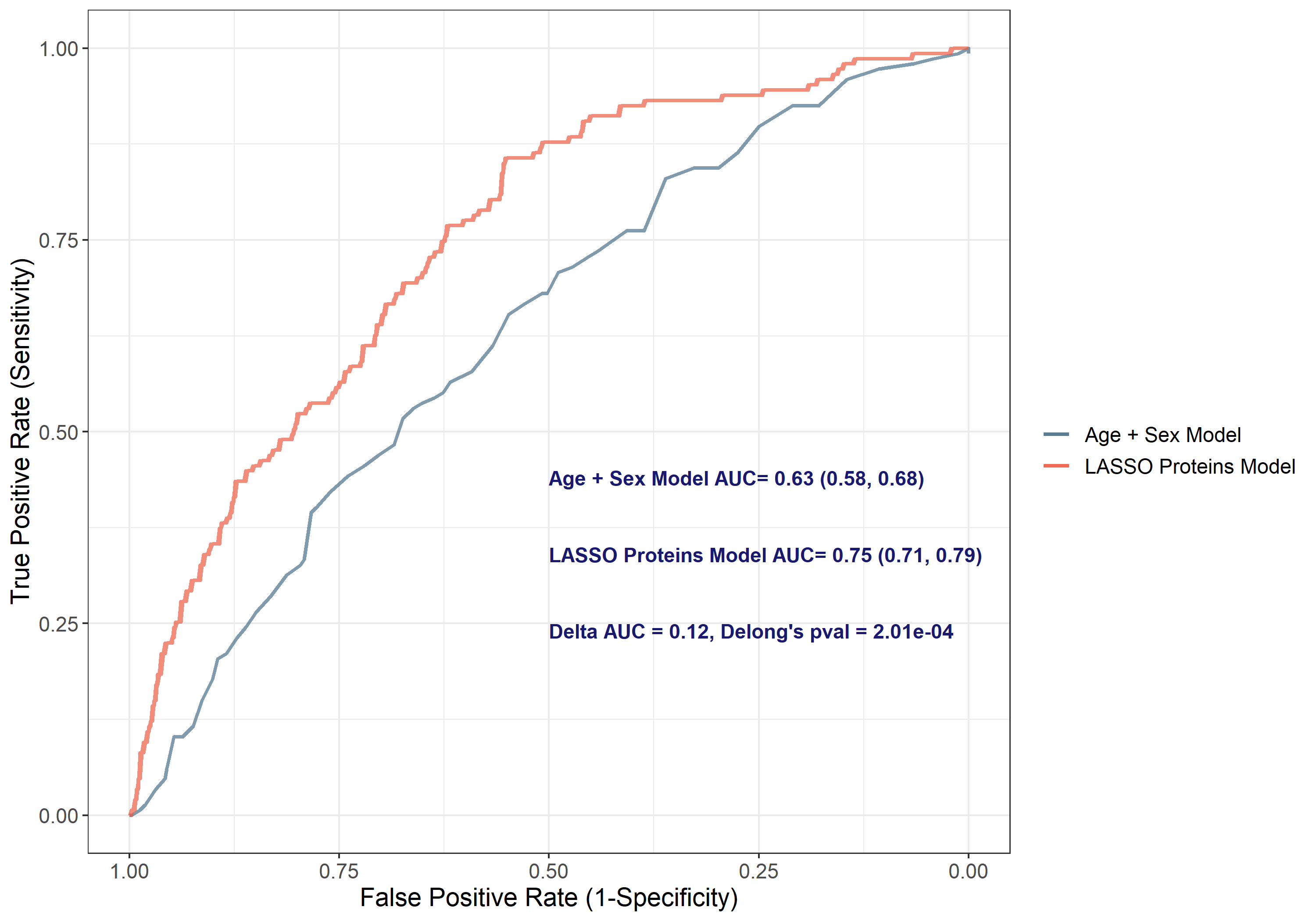


Figure S2: ROC curve comparing the bootstrap ranking LASSO selected protein model with age and sex model predicting incident MetS in KORA, showing the AUCs, their 95% CI and the difference (delta AUC) and p-value of the DeLong test comparing both models.


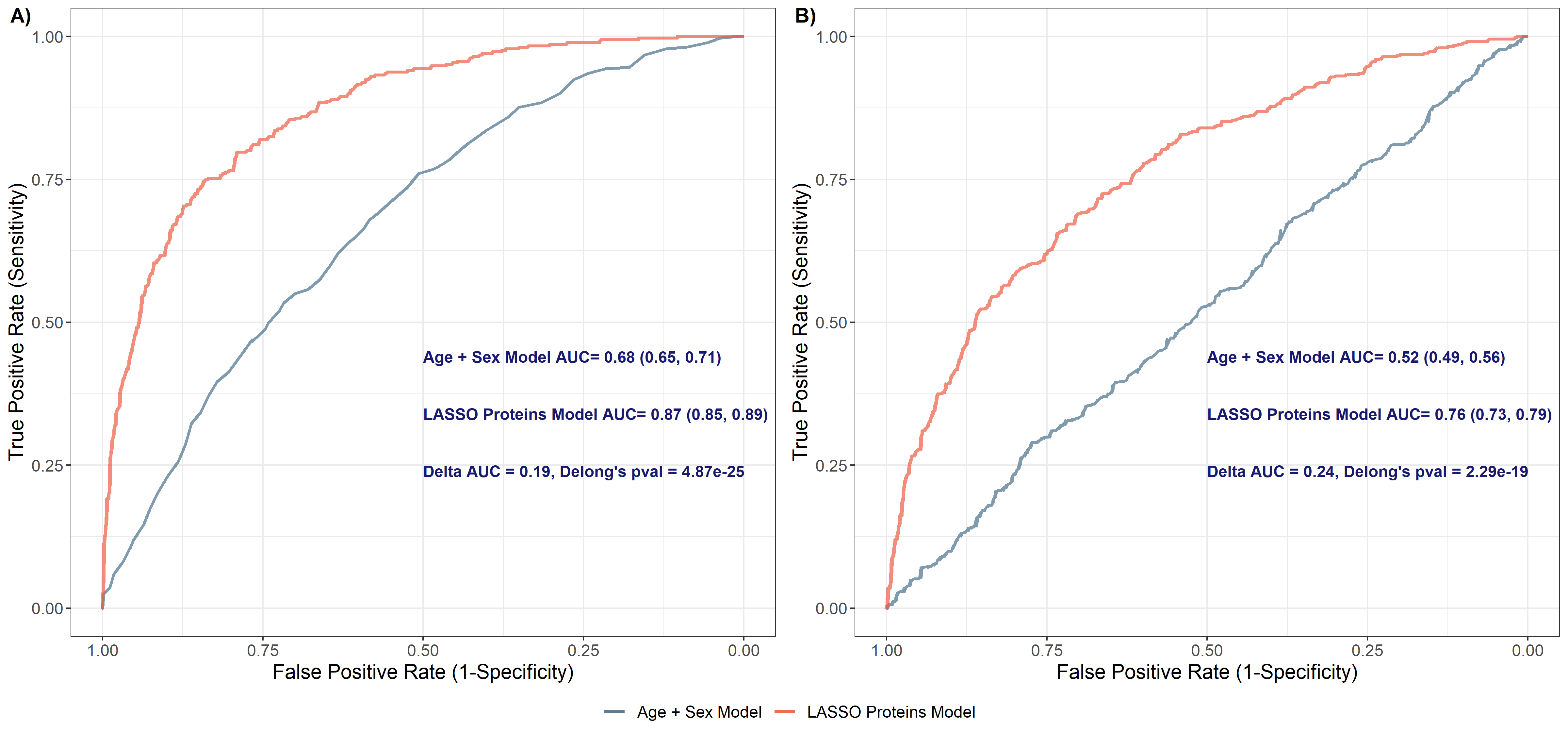


Figure S3: ROC curve comparing the bootstrap ranking LASSO selected protein model with age and sex model predicting prevalent MetS in KORA (A) and HUNT (B), showing the AUCs, their 95% CI and the difference (delta AUC) and p-value of the DeLong test comparing both models.


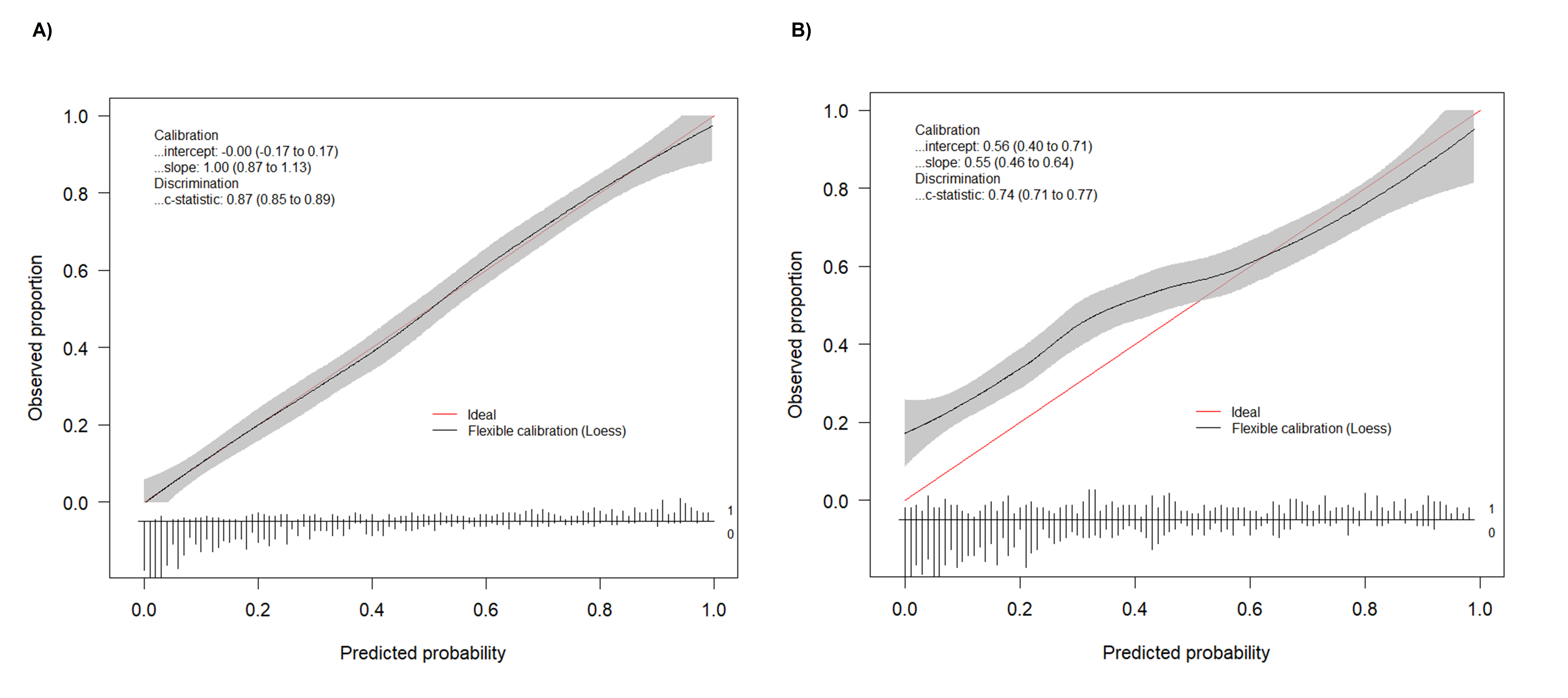


Figure S4: Calibration plots of the bootstrap ranking LASSO-selected MetS diagnostic model in: A) KORA; B) HUNT.


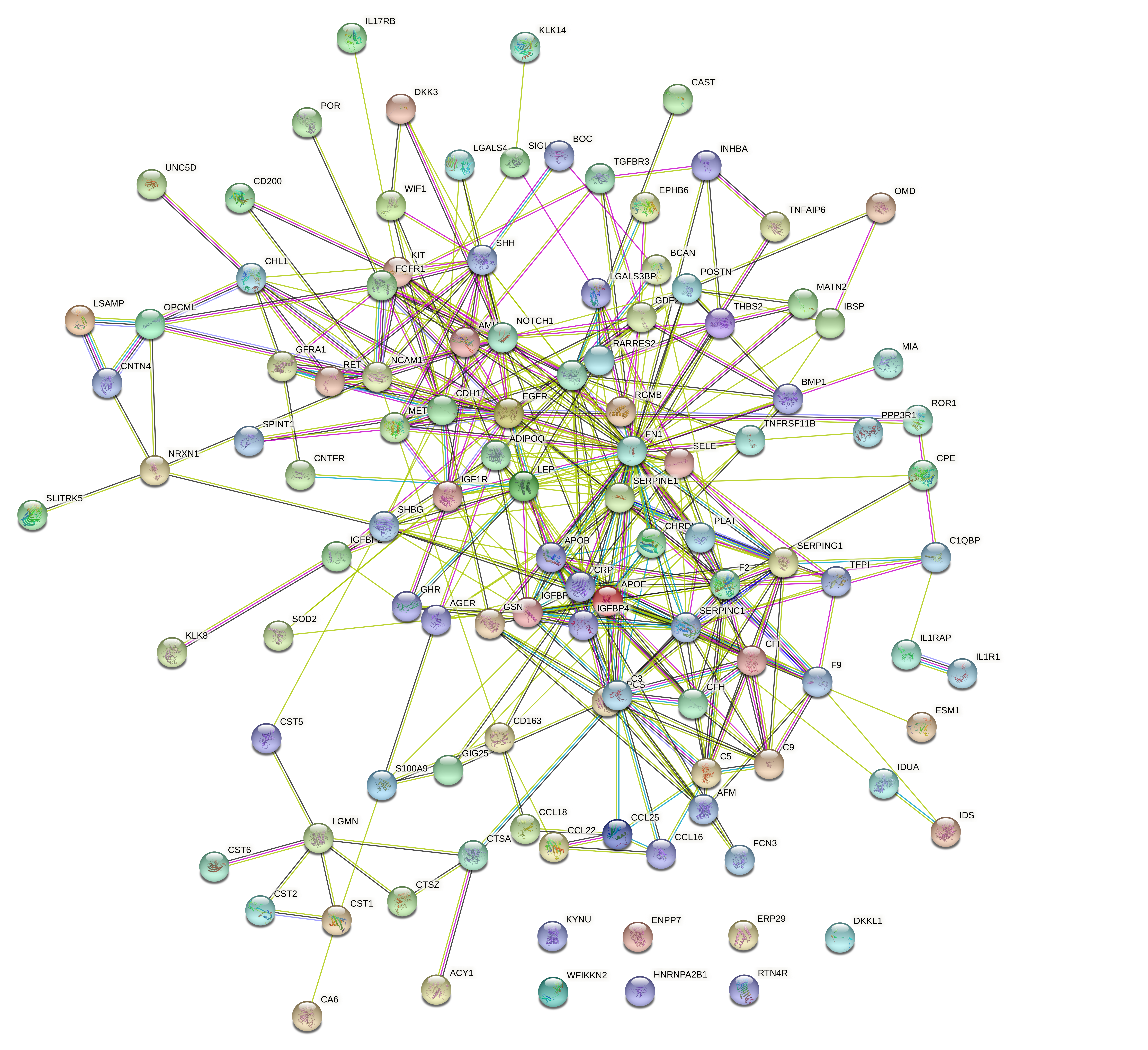


Figure S5: STRING protein-protein interaction network constructed using the prevalent or incident MetS associated proteins in KORA without adding additional interactor proteins.

References

1. Szklarczyk D, Gable AL, Lyon D, Junge A, Wyder S, Huerta-Cepas J, et al. STRING v11: protein-protein association networks with increased coverage, supporting functional discovery in genome-wide experimental datasets. Nucleic Acids Res. 2019;47(D1):D607-d13.
